# Editorial: Microbial biotransformation of natural flavor compounds

**DOI:** 10.3389/fmicb.2023.1243194

**Published:** 2023-07-11

**Authors:** Giacomo Zara, Gang Fan

**Affiliations:** ^1^Department of Agricultural Sciences, University of Sassari, Sassari, Italy; ^2^Key Laboratory of Environment Correlative Dietology, Ministry of Education, College of Food Science and Technology, Huazhong Agricultural University, Wuhan, China

**Keywords:** natural flavor compounds, biotransformation, starter selection, food and feed fermentation, valorization of food wastes

Aroma and taste are among the main components of foods and pharmaceuticals that attract consumers as they influence the sensory properties of the product. Most flavor compounds are produced by chemical processes such as *de novo* synthesis or extraction/purification from fruits, vegetables, and animal matrixes. Biotechnological processes based on the activity of microbial cells or microbial extracts provide a more natural alternative to produce aroma compounds. Microbial flavor compounds are increasingly popular because of the growing consumer demand for sustainable and natural food additives. Indeed, microbial biotransformation has long been proven to be a highly efficient technology with the advantages of reaction specificities and environmental friendliness. There are also documented health benefits deriving from microbial activity, such as the production of molecules with antimicrobial, functional, and probiotic properties. Given all these advantages, there is an increasing need to screen and explore new microorganisms and investigate the metabolic pathways involved in the microbial production of flavor compounds. Furthermore, it is important to implement technologies to improve the efficiency of biological transformation by cloning, expression, and purification of key microbial enzymes, or by developing high-efficiency microbial cell factories. In this context, this Research Topic aims to provide an overview of the recent studies on the microbial biotransformation of natural flavor compounds, focusing on the optimization of fermentation processes by properly exploiting natural as well as engineered microbial strains, or by developing novel additives and inoculation protocols.

This Research Topic comprises five original research articles, contributed by 43 authors.

Li M. et al. analyzed the effect of different additives on the chemical composition and fermentation quality of a novel animal protein feed made from paper mulberry (*Broussonetia papyrifera* L.). Particularly, the authors found that formic acid, *Amomum villosum* essential oil, and lactic acid bacteria altered the bacterial community structure during paper mulberry ensiling, resulting in improved silage quality. In addition to animal feeding, the results obtained have a broader interest considering that the use of the *A. villosum* essential oil increased the abundance of *Lactococcus, Levilactobacillus*, and *Lentilactobacillus*, which are pro-technological bacteria involved in many different fermentation processes, such as those required to produce dairy, meat, and vegetable foods and feeds.

Li P. et al. dealt with the effect of different inoculation protocols on the flavor quality of a traditional Chinese alcoholic beverage (*Black Huangjiu*). It was found that compared with the traditional fermentation, the tested sequential inoculation protocol increased non-volatile flavor compounds (organic acids and derivatives) and decreased lipids and lipid-like molecules. Therefore, the beverage fermented by sequential inoculation had a stronger, sweeter, mellow, and softer taste. Metabolic changes were correlated with variation in the relative abundances of *Saccharomyces* and particularly *Rizhopus*, which was found at higher populations in the sequential protocol. These results agree with previous studies carried out on fermented beverages, such as wine and beer, that showed the uttermost importance of the proper inoculation method to enhance the contribution of different yeast species in the development of the sensorial properties of the final product.

On this matter, Wang et al. assessed the dynamics of bacterial communities, their metabolic pathways, and flavor production during the ripening of a fermented sausage inoculated with starter cultures of *Lactiplantibacillus plantarum* and *Staphylococcus xylosus*. Overall, the bacterial abundance and metabolic activity in the sausage increased during the maturation process, with *Lactiplantibacillus, Staphylococcus, Lactococcus, Leuconostoc*, and *Weissella* the most abundant genera in all phases of fermentation. Furthermore, the authors showed that the presence of *Staphylococcus* and *Leuconostoc* is related to the formation of flavor compounds. These results indicate that inter-species interactions between exogenous microbial inoculants and the resident microbiota should be considered among the (side) effects of microbial starters. As shown by the authors, the perturbation of the resident microflora due to starter addition resulted in the dominance of endogenous microbial genera (in their case *Leuconostoc*), with a significant effect on the overall flavor profile of the final product.

The significance of proper starter selection for the improvement of the fermentation process is the main aim of the study by Chen et al.. The authors engineered a strain of *Yarrowia lipolytica* to increase the production of the aroma compound β-ionone from the fermentation of food waste and sugarcane bagasse. Particularly, the activity of this engineered yeast resulted in the highest yield reported to date for β-ionone from the fermentation of organic waste. The authors suggested that scaling up the described integrated process could lead to the economical large-scale conversion of inedible food and agricultural waste into valuable aroma compounds. In this respect, food consumption and waste generation are expected to increase as the world population increases. Thus, developing protocols and technologies for the proper exploitation and valorization of food waste is a current key topic of global research and the cornerstone of the circular economy.

The importance of the proper monitoring of fermentation processes when scaling up from laboratory to factory conditions was highlighted by Li H. et al., where the authors showed that koji samples had a greater number of fungi when produced at a large scale than when produced in laboratory conditions. In addition, the chemical and volatile composition of the product was more influenced by the fermentation conditions than by the starter strain used. The authors showed that the chemical and biochemical differences observed in koji samples made with two different starters at laboratory conditions were not detected under factory conditions. This suggests that the development of an efficient fermentation process that enables the large-scale biosynthesis of aroma molecules remains a challenge.

Hence, to conclude the whole Research Topic, the contributions received highlighted the importance of proper strain selection and process optimization for the suitable production of aroma compounds through bioprocesses, and we are confident that the articles included in this Research Topic will inform the scientific community about the current knowledge and challenges that still must be overcome in this field.

## Author contributions

All authors listed have made a substantial, direct, and intellectual contribution to the work and approved it for publication.

